# A tale of two CETs: Untangling cyclic electron transfer

**DOI:** 10.1093/plphys/kiad088

**Published:** 2023-02-14

**Authors:** Gustaf E Degen

**Affiliations:** Plants, Photosynthesis and Soil, School of Biosciences, University of Sheffield, Firth Court, Western Bank, Sheffield, S10 2TN, UK

Cyclic electron transfer (CET) is a bypass of the normal Z-scheme of the photosynthetic light reactions that allows energy that otherwise would have gone into making NADPH to be diverted toward making more ATP. There are, in fact, 2 competing forms of CET, but before delving into the specifics of these processes, a quick primer on chloroplast electron flow may be useful.

Photosynthesis converts the energy from sunlight into chemical energy to assimilate CO_2_ from the atmosphere into organic compounds. Light energy is harvested by PSII in chloroplasts and is transferred to electrons that travel along the linear electron transfer (LET) chain in the thylakoid membrane. Plastoquinone (PQ) accepts electrons from PSII, resulting in the formation of plastoquinol (PQH_2_). Electrons from PQH_2_ are then transferred to plastocyanin via the cytochrome b_6_f complex (cyt*b_6_f*) to reduce PSI. Electrons are excited again in PSI and are finally transferred to Ferredoxin (Fd), which is used by ferredoxin-NADP^+^ reductase to reduce NADP^+^ to NADPH (reviewed in Johnson 2016). During this process, protons are transferred by PSII and the cyt*b_6_f* complex from the chloroplast stroma to the lumen, generating proton motive force (pmf) that is used by ATP synthase to generate ATP.

The ATP/NADPH ratio produced by LET, however, is insufficient to meet the demands of CO_2_-fixing reactions, and this is where the 2 types of CET come into play: the CETs associated with PSI increase the ATP/NADPH ratio by generating additional pmf, producing ATP without NADPH (Yamori et al. 2015). The PROTON GRADIENT REGULATION 5 (PGR5)-dependent pathway is the major CET route in plants (Munekage et al. 2002, 2004). Here, electrons are transferred back to cyt*b_6_f*, involving the still somewhat elusive PGR5 protein, which results in extra proton transfer to the lumen. There is, however, a secondary CET pathway that uses the NADH dehydrogenase-like (NDH) complex to accept electrons from Fd and to pump protons into the thylakoid lumen. Although conserved in plants, the NDH route is only important at low light, as shown in studies using the NDH-deficient *chlororespiratory reduction* (*crr*) mutants (Yamori and Shikanai 2015; Yamori et al. 2015).

**Figure 1.**
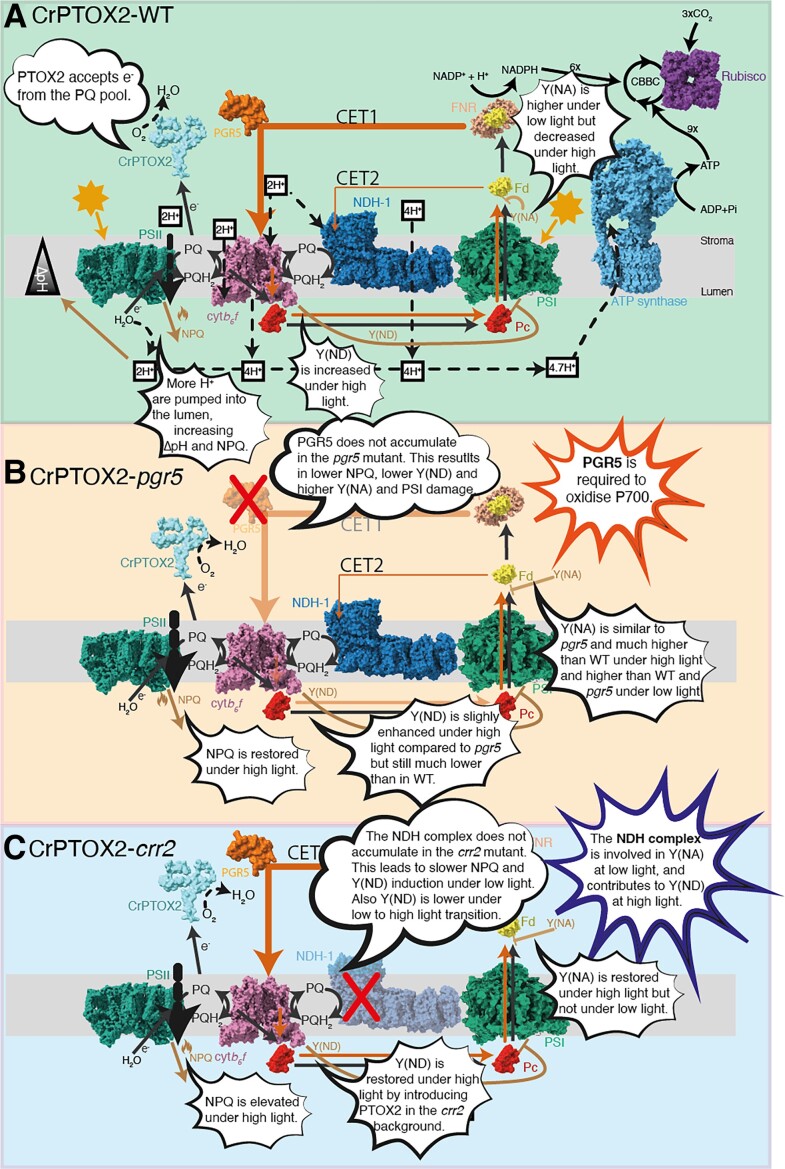
Impact of CrPTOX2 on photosynthesis in WT and CET mutants. A) CrPTOX2 oxidizes the PQH_2_ pool and reduces oxygen to water in the WT. This drives increased proton transfer into the thylakoid lumen, increasing ΔpH and nonphotochemical fluorescence quenching (NPQ). This also increases photosynthetic control (Y(ND)) under high light. Acceptor-side limitation (Y(NA)) is increased under low light but decreased under low light. B) In the CrPTOX2-*pgr5* line, PGR5-dependent CET is abolished. PTOX2 restores NPQ under high light but not Y(ND). Y(NA) in the CrPTOX2-*pgr5* line is similar to that of *pgr5* under high light, suggesting that PGR5 is required for P700 oxidation. C) In the NDH-deficient *crr2* mutant, CrPTOX restores NPQ, Y(ND), and Y(NA) under high light. However, Y(NA) is not restored under low light, suggesting that NDH is required for Y(NA) under low light. Protein structures were obtained from https://www.rcsb.org. CET, cyclic electron transfer; CBBC, Calvin–Benson–Bassham cycle; cyt*b_6_f*, cytochrome *b_6_f* complex; Fd, ferredoxin; FNR, ferredoxin-NADP^+^ reductase; Pc, plastocyanin; PQ, plastoquinone; PQH_2_, plastoquinol; PGR5, PROTON GRADIENT REGULATION 5; NDH-1, NADH dehydrogenase-like complex1; Rubisco, Ribulose-1,5-bisphosphate carboxylase-oxygenase.

Because the rates of CET are regulated by donor- or acceptor-side limitations, light levels play a key role in determining CET activities. Under excessive light, CET pumps additional protons into the lumen, lowering the pH and triggering nonphotochemical fluorescence quenching (NPQ) and slowing down electron transfer at the cytb_6_f complex; excessive light, therefore, causes donor-side limitation to PSI (Fig. 1).

Furthermore, PGR5 is required to oxidize P700 in PSI, thus preventing acceptor-side limitation that would lead to overreduction and damage of PSI (Zhou et al. 2022). As expected, under high light intensities, the *pgr5* mutant is characterized by low NPQ and high acceptor-side limitation. Plastid terminal oxidase (PTOX) provides another safety valve for electrons by accepting electrons from PQH_2_ and reducing oxygen to water. In the model species *Arabidopsis thaliana* (Arabidopsis), PTOX is only important during chloroplast development, but it plays a much more important role in *Chlamydomonas reinhardtii* (Peltier and Cournac 2002). Previous work has revealed that by introducing *C. reinhardtii* PTOX (CrPTOX) into the *pgr5* mutant, the function of PGR5 could be complemented, except for the oxidation of P700 (Zhou et al. 2022). This indicates that PGR5 is required to move electrons from the acceptor-side of PSI to prevent overreduction. A remaining question is the extent to which the NDH pathway contributes to acceptor-side regulation.

In this issue of *Plant Physiology*, Zhou et al. (2023) introduced CrPTOX into the NDH-deficient *crr2* mutant to disentangle the contribution of both CET pathways to P700 oxidation. After verifying the accumulation of CrPTOX in the wild-type (WT), *crr2*, and *pgr5* backgrounds, the authors assessed the effect this had on photosynthesis during increasing light levels. NPQ was mildly enhanced in the CrPTOX2-*crr2* lines at low light but was unaffected in the *crr2* background itself. Surprisingly, the 1-qL parameter (a measure of acceptor-side limitation of PSII) was slightly enhanced in *crr2* and CrPTOX-*crr2* at lower light, suggesting a reduced PQ pool.

Due to the lack of the NDH complex in *crr2* and CrPTOX2-*crr2*, the PQ pool should be oxidized because electrons are not routed back via NDH. The authors were able to link this to the delayed induction of state transitions, which occur when PSII moves electrons faster than PSI, leading to a reduced PQ pool. This induces the phosphorylation of light-harvesting complex II (LHCII) via the LHCII kinase STATE TRANSITION 7 (STN7). LCHII then migrates to PSI to increase absorption of light so that more electrons can flow from the PQ pool to PSI. In the *stn7* mutant, which cannot induce state transitions, the 1-qL parameter was much higher in low light, suggesting that the PQ pool was more reduced. Thus, the authors concluded that the reduced PQ pool in *crr2* and CrPTOX-*crr2* was in part related to disturbed state transitions.

In a previous study, the authors showed that CrPTOX2 in the *pgr5* background efficiently operated as a safety valve for electrons during photosynthetic induction. A similar observation was made for the CrPTOX-*crr2* lines in this study, where NPQ was induced faster. Furthermore, donor-side limitation was much higher after onset of light in the CrPTOX-*crr2* lines because the thylakoid lumen was more acidified due to extra proton pumping, which was also confirmed by increased pmf, and suggested that CrPTOX2 operated efficiently only during early induction of photosynthesis. This is because the Calvin–Benson–Bassham cycle (the CO_2_-fixing reactions) is not yet activated and thus is a limiting factor immediately after transitioning from dark to light. A similar electron sink limitation occurs during fluctuating light (as experienced by plants throughout the day), where light intensity shifts drastically within seconds to minutes.

To simulate fluctuating light, plants were exposed to 3 cycles of low light for 5 min and high light for 1 min. During the high light periods, the CrPTOX2-*crr2* lines had higher an NPQ and were able to induce photosynthetic control faster than WT. Thus, CrPTOX2 was able to restore oxidation of P700 in the high light periods in the *crr2* background, but not in the low-light periods. This suggests that the NDH complex contributes to P700 oxidation in low-light periods. Overall, accumulation of CrPTOX2 in the *crr2* background prevented PSI photodamage during fluctuating light, in contrast to CrPTOX2-*pgr5* lines. The authors concluded that the NDH complex is involved in acceptor-side regulation at low light alongside PGR5, but its contribution at high light is negligible. However, both CET pathways contributed to photosynthetic control at high light.
